# The Prevalence and Correlates of Diabetes Distress among South Asians Living in New York City (NYC): Baseline Results from a Randomized Trial

**DOI:** 10.21203/rs.3.rs-2806895/v1

**Published:** 2023-06-08

**Authors:** Farhan Mohsin, Laura Wyatt, Hayley Belli, Shahmir Ali, Deborah Onakomaiya, Supriya Misra, Yousra Yusuf, Shinu Mammen, Jennifer Zanowiak, Sarah Hussain, Haroon Zafar, Sahnah Lim, Nadia Islam, Naheed Ahmed

**Affiliations:** NYU Grossman School of Medicine; NYU Grossman School of Medicine; NYU Grossman School of Medicine; NYU Grossman School of Medicine; NYU Grossman School of Medicine; San Francisco State University; NYU Grossman School of Medicine; NYU Grossman School of Medicine; NYU Grossman School of Medicine; NYU Grossman School of Medicine; NYU Grossman School of Medicine; NYU Grossman School of Medicine; NYU Grossman School of Medicine; NYU Grossman School of Medicine

**Keywords:** South Asian, immigrants, type 2 diabetes, diabetes distress, mental health, immigrant health, health disparities

## Abstract

**Background:**

Type 2 diabetes (T2D) disproportionately affects South Asians in the United States (US). Living with T2D can be challenging due to the distress it can create for an individual. Distress associated with diabetes, commonly known as diabetes distress (DD), may lead to complications and challenges with the management of diabetes. This study aims to describe the prevalence of DD among a sample of South Asians in New York City (NYC) seeking care in community-based primary care settings and its association with sociodemographic characteristics and clinical measures.

**Methods:**

This study utilized baseline data from the Diabetes Research, Education, and Action for Minorities (DREAM) Initiative, an intervention designed to reduce hemoglobin A1C (HbA1c) among South Asians with uncontrolled T2D in NYC. DD was measured using the Diabetes Distress Scale (DDS). First, descriptive statistics were used to analyze sociodemographic variables. Chi-square tests assessed categorical variables and Wilcoxon Rank Sum tests assessed continuous variables using a Type I error rate of 0.05. Logistic regression was performed to determine if HbA1c and mental health, along with other covariates, were associated with dichotomized DDS subscales.

**Results:**

Overall, 415 participants completed the DDS at baseline. Median age was 56 years (IQR: 48-62). A total of 25.9% had high emotional burden distress, 6.6% had high physician-related distress, and 22.2% had high regimen-related distress based on subscales. In adjusted analyses, individuals with any days of poor mental health had significantly higher odds of overall distress (OR:3.7, p=0.014), emotional burden distress (OR:4.9, p<0.001), and physician-related distress (OR:5.0, p=0.002) compared to individuals with no days of poor mental health. Individuals with higher HbA1c had significantly higher odds of regimen-related distress (OR:1.31, p=0.007).

**Conclusions:**

Findings suggest that DD is prevalent among this sample of South Asians with diagnosed T2D in NYC. Screening for DD in patients with prediabetes/diabetes should be considered by providers to help provide mental and physical health services during primary care visits. Future research can also benefit from a longitudinal analysis of the impact of DD on diabetes self-management, medication adherence, and mental and physical health.

**Trial registration:**

This study uses baseline data from “Diabetes Management Intervention For South Asians” (NCT03333044), which was registered with clinicaltrials.gov on 6/11/2017.

## Background

Type 2 diabetes (T2D) is a leading cause of mortality and morbidity in the United States (US) and disproportionately affects South Asians (Asian Indians, Bangladeshis, Bhutanese, Nepali, Pakistanis, and Sri Lankans) in the US (1–3), one of the fastest-growing ethnic minorities in the US and New York City (NYC) (4,5). Findings from the 2011–2016 National Health and Nutrition Examination Survey (NHANES) revealed that South Asians had the highest prevalence of diabetes (23.3%) when compared to non-Hispanic Whites (12.1%) and non-Hispanic Asians overall (19.1%) (6). Data from the 2009 to 2012 Racial and Ethnic Approaches to Community Health (REACH) US Risk Factor Survey had similar findings; the age-adjusted prevalence of diabetes was highest among Asian Indians (19.0%) compared to Black, Korean, and Chinese groups (7). Results from the 2014–2018 NYC Community Health Survey (NYC CHS) found that Asian Indians (21%) and underrepresented South Asians such as Bangladeshi, Bhutanese, Nepali, Pakistani, and/or Sri Lankan (15%) had the highest prevalence of self-reported diabetes compared to the overall NYC sample (11%) (8). Finally, the 2004 NYC Health and Nutrition Survey (NYC HANES) found a higher diabetes prevalence among foreign-born South Asians (35.4%), compared to Whites (10.8%) and overall Asians (16.1%) (9).

Living with T2D can be challenging due to the unique psychological impact the illness can have on an individual’s mental health (10). Distress associated with diabetes may lead to complications and challenges with the management of diabetes (11–13), which may ultimately worsen diabetes-related outcomes. Diabetes distress (DD) is defined as the distinct emotional burdens or worries an individual may experience while trying to manage and live with diabetes (14). Symptoms of DD may include feeling discouraged, worried, frustrated or tired of dealing with diabetes care or feeling as if diabetes is controlling oneself instead of the other way around (15).

The prevalence of DD is common and can last over time (16). High levels of DD have been linked to low diabetes self-efficacy, poor glycemic control, and poor quality of life (17). High DD levels are also associated with poor diet, high blood pressure (BP), lower medication adherence, and low physical activity (18). Among individuals living in low- and middle-income South Asian countries, a scoping review identified a high prevalence of DD (18%-76.2%), where high levels of DD were associated with lower medication adherence (19). Cross-sectional studies from South Asian countries such as Bangladesh (52.5%) and Pakistan (76.2%) also reported high levels of DD, with the study in Pakistan identifying lower education and income to be significantly associated with DD (17,20). Mental illnesses, such as depression, have been linked with DD (20,21); nearly a third of participants in the Bangladesh study were found to have both DD and depression (20). A cross-sectional study among South Asians in Canada also found a high prevalence of DD (52.5%), with total DD having a moderately positive correlation with depression (r = 0.70, p < 0.001) (21).

Much of the existing literature on DD among South Asians has come from studies based in Canada and South Asian countries. To our knowledge, this is the first study focusing on DD among South Asians in the US with T2D. Combined with the rise of pre-diabetes in the US (22), there may be significant differences in socio-demographics and access to healthcare among South Asians in the US in comparison to other countries, making it of paramount importance to examine the impact of DD on South Asian Americans. This study aims to examine the prevalence of DD among South Asians in NYC and its associations across demographic and health-related factors.

## Methods

### Study population

This study utilized baseline data from the randomized treatment group of the Diabetes Research, Education, and Action for Minorities (DREAM) Initiative, a multilevel diabetes management intervention designed to improve hemoglobin A1C (HbA1c) among South Asian individuals (from Bangladesh, Pakistan, India, Guyana, Nepal, and Trinidad) at small primary care practices (PCPs) in NYC with uncontrolled T2D (23). Study recruitment protocols have been previously described (23); briefly, individuals with 1) HbA1c levels > 7.0% in the past 12 months; 2) a visit to the provider’s office in the last 12 months; 3) between 21–74 years old; and 4) not pregnant at the time of screening were considered eligible to participate (23). Lists of eligible participants were generated from electronic health record (EHR) patient lists at participating PCPs. Patients who were randomized into the treatment group were contacted by community health workers (CHWs) and invited to participate in the study. Control group individuals were not contacted. The study took place over three years from 2019 to 2022, with three intervention waves. Each wave took place over six months and consisted of five monthly, CHW-led sessions lasting 90–120 minutes. Baseline data were collected between July 2019 and August 2022. A total of 419 individuals were enrolled in the treatment group, and 415 of these individuals completed the Diabetes Distress Scale questionnaire at baseline and were included in this analysis. Written informed consent was obtained from all treatment group participants. The NYU Langone Health IRB ethics committee approved the study. Further details are described in the study protocol (23).

### Measures

#### Diabetes Distress.

DD was measured using the Diabetes Distress Scale (DDS) (14). The DDS has been shown to have good internal reliability and validity and can serve as a tool to measure DD for research purposes to examine relationships between DD and blood glucose levels, diet, physical activity, and self-efficacy in the management of diabetes (Cronbach’s alpha = 0.93, with subscale ranging from 0.88–0.90) (18,24). Psychometric validation of the DDS among South Asian subgroups such as Bangladeshis has also found the scale to be a valid and reliable tool to assess distress among Bangladeshis living with T2D (Cronbach’s alpha = 0.84, with subscales ranging from 0.70–0.88 and the lowest score seen in physician-related distress) (10).

The original DDS consisted of four subscales and 17 questions total: Emotional Burden (5 questions), Physician-related Distress (4 questions), Regimen-related Distress (5 questions), and Interpersonal Distress (3 questions). After the second wave of baseline data collection, CHWs reported that the questionnaire was long and burdensome to complete for participants. The questions were assessed by the study statistician, and one scale (Interpersonal Distress - Cronbach’s alpha = 0.882) was removed, as well as two questions from the Emotional Burden subscale and one question from Regimen-related Distress subscale. No questions were removed from the Physician-related distress subscale. Interpersonal distress was removed due to no difference in the subscale at baseline and follow-up for the first study wave. The Cronbach’s alpha for two waves of data collection with all questions was 0.940, while the Cronbach’s alpha for the reduced scale over three waves of data collection was 0.918. Cronbach’s alphas for each final subscale are: Regimen-related Distress: 0.894; Emotional Burden subscale: 0.916; and Physician-related distress: 0.821. See Supplementary File 1.

Respondents were asked to “Consider the degrees to which each item may have distressed or bothered you during the past month.” Responses for the DDS questions included: Not a problem (1), slight problem (2), moderate problem (3), Somewhat serious problem (4), serious problem (5), and very serious problem (6). The mean of the questions was calculated for each subscale and all questions (the overall scale) ranged from 1–6. Informed by past research where a mean score of ≥ 3 was the threshold for being distressed, a dichotomous outcome was calculated for each subscale: low DD: <3 and high DD: ≥3 (25,26).

#### Healthy days.

Two questions from the CDC’s Core Healthy Days Measures were used to measure healthy days: “Now thinking about your physical health, which includes physical illness and injury, for how many days during the past 30 days was your physical health not good?” and “Now thinking about your mental health, which includes stress, depression, and problems with emotions, for how many days during the past 30 days was your mental health not good?” with responses ranging from 0–30 (27). Each question was dichotomized into no days (0) and any days (1 – 30) (27).

#### Emotional support.

The 4-item PROMIS Emotional Support (4a) was used to assess perceived feelings of being cared for and valued and having confident relationships (28). The raw total score ranges from 4 to 20; a t-score was calculated, ranging from 25.7 to 62.2, with a higher score representing higher levels of support (28).

#### Clinical measures.

Systolic blood pressure (SBP), diastolic blood pressure (DBP), weight, BMI, and HbA1c were obtained from EHRs at each PCP site. All clinical measures were analyzed as continuous variables, and one categorical threshold was constructed for BP control: <140/90 (29–31).

#### Socio-demographics.

Socio-demographic variables included sex (male vs. female), age (continuous), country of birth (Bangladesh vs. other [Pakistan, India, Guyana, Nepal, Trinidad]), years living in the US (continuous), marital status (married vs. not married), level of education (< high school, high school/some college, and college graduate), and English spoken fluency (very well/well vs. not well/not at all). Age and sex were obtained from the EHR records used for eligibility. All other measures were obtained from the baseline surveys collected by the CHWs.

### Statistical analysis

Data were analyzed using SPSS version 28.0. Descriptive statistics were analyzed at baseline overall and stratified by sex. Differences in sex were assessed using Pearson Chi-square tests for categorical variables and Wilcoxon Rank Sum tests for continuous variables. A type I error rate threshold of 0.05 was used to assess significance with no correction for multiple comparisons as all analyses were exploratory. Logistic regression was then performed to determine if HbA1c and mental health, along with other covariates, were associated with DDS subscales first in unadjusted, univariable logistic regression. Variables found to be significant at p < 0.20 in the unadjusted, univariable regression were considered, and final adjusted models were fit to include variables significant at a type I error rate threshold of 0.05. Backwards stepwise selection was used to eliminate non-significant covariates in the final model. All final adjusted models included age, sex, education, and years in the US, regardless of significance. Odds ratios (ORs), 95% confidence intervals (CIs), and p-values were calculated.

## Results

Table 1 presents socio-demographic, scale variables, and clinical measures overall and stratified by sex (n = 415). Median age was 56.0 years (Interquartile range [IQR] = 48.0–62.0) and the majority were married (93.2%). All individuals were born outside of the US. While the study focus was on South Asian individuals in NYC, the study sample was primarily composed of individuals born in Bangladesh (69.4%), followed by India (14.6%). In this sample, 25.9% reported high emotional burden distress, 6.6% high physician-related distress, and 22.2% high regimen-related distress.

When stratifying by sex, men were significantly more likely than women to have graduated from high school (51.5% vs. 23.3%, p < 0.001), to speak English very well or well (54.1 % vs. 27.1 %, p = < 0.001), and to have lived in the US for longer (Median: 15.0 years [IQR = 8.0, 22.8] vs. 11.0 years [IQR = 5.6, 19.0], p = < 0.001). Women were significantly more likely to have any days of poor mental or physical health (p = 0.007 and p = 0.004, respectively), and a higher emotional support t-score (p < 0.001). While women had a significantly higher median score for all DDS subscales, only the dichotomized DDS Overall scale and regimen-related distress subscale were significantly different by sex.

Univariate regression analyses found differences in dichotomized DDS subscales (p < 0.20) for the following scales and variables: DDS overall (sex, age, education, marital status, English fluency, country of birth, years in the US, mental health days, emotional support t-score, HbA1c, and SBP); emotional burden subscale (sex, age, education, English fluency, country of birth, years in the US, mental health days, emotional support t-score, HbA1c, and SBP); physician-related distress subscale (education, country of birth, mental health days, physical health days, emotional support t-score, DBR and BP control); and regimen-related distress (sex, education, English fluency, country of birth, years in the US, emotional support t-score, BMI, weight, HbA1c, and SBP) (Table 2). These variables were then each tested for inclusion in a final adjusted regression model, as applicable for the outcome.

After conducting adjusted regression analyses (Table 3), we found the following results:

### DDS Score - Overall

Individuals born in Bangladesh had significantly lower odds of overall distress (OR = 0.1, 95% CI: 0.0, 0.3, p < 0.001) compared to individuals born in other South Asian countries, individuals with any days of poor mental health in the past month had higher odds of overall distress (OR = 3.7, 95% CI: 1.3, 10.4, p = 0.014) compared to individuals with no days of poor mental health, and individuals with lower emotional support had significantly higher odds of overall distress (OR = 0.92, 95% CI: 0.88, 0.96, p < 0.001) compared to individuals with high emotional support.

### DDS score - Emotional Burden

Individuals born in Bangladesh had significantly lower odds of emotional burden (OR = 0.1, 95% CI: 0.0, 0.1, p < 0.001) compared to individuals born in other South Asian countries; individuals with any days of poor mental health in the past month had significantly higher odds of emotional burden (OR = 4.5, 95% CI: 1.5, 13.6, p < 0.001) compared to individuals with no days of poor mental health; finally individuals with less than high school education had significantly higher odds of emotional burden (OR = 4.5, 95% CI: 1.5, 13.6, p = 0.007) compared to college graduates.

### DDS score – Physician-Related Distress

Individuals born in Bangladesh had significantly lower odds of physician-related distress (OR = 0.3, 95% CI: 0.1, 0.8, p = 0.015) compared to individuals born in other South Asian countries, and individuals with any days of poor mental health in the past month had significantly higher odds of physician-related distress (OR = 5.0, 95% CI: 1.8, 13.7, p = 0.002) compared to individuals with no days of poor mental health.

### DDS score – Regimen-Related Distress

Individuals born in Bangladesh had significantly lower odds of regimen-related distress (OR = 0.2, 95% CI: 0.1, 0.3, p < 0.001) compared to individuals born in other South Asian countries, individuals with lower emotional support had significantly higher odds of regimen-related distress (OR = 0.95, 95% CI: 0.92, 0.99, p = 0.006) compared to individuals with high emotional support, and individuals with higher HbA1c had significantly higher odds of regimen-related distress (OR = 1.31, 95% CI: 1.08, 1.60, p = 0.007) compared to individuals with lower HbA1c.

## Discussion

In our sample of South Asians with T2D in NYC, 15.9% reported having high overall diabetes distress. Previous studies in Bangladesh, Canada, China, Malaysia, and Saudi Arabia have all reported different prevalence of DD (25.0-49.2%) (32–36). Meta-analyses of the prevalence of DD in people with T2D in the US also reveal a wide range of prevalence of DD (19-79.5%) (37). Findings from such studies indicate that the prevalence of DD may vary greatly across different countries and healthcare settings. The discrepancy between the DD prevalence in previous studies compared to this study may result from methodological differences in the study design, data collection, or analysis methods. It is also likely that the study population is composed of individuals who receive good diabetes care because participation in the study required participants to have visited their provider at least once in the past 12 months, which may explain the lower observed prevalence of DD in this study compared to other studies. These discrepancies may also be because the samples for these studies were drawn from populations with different demographic characteristics and cultural backgrounds.

A significantly greater proportion of females in our sample had high DD when compared to males. The Saudi Arabia study reported similar findings in that females were significantly more likely to have high DD compared to males (36). Indeed, previous studies have reported that there may be a link between being female and the persistence of DD (37,38). The increased persistence of DD among females with T2D may be attributable to sex-based differences: psychological stress among females is higher compared to males (39), with females encountering more stressful life events and being more negatively impacted by stressful life events when compared to males (40,41).

Individuals born in Bangladesh had lower odds of overall DD as well as lower odds of emotional burden, physician-related distress, and regimen-related distress compared to individuals born in other South Asian countries. Growing evidence suggests that a network of community- or family-level factors may improve the health outcomes of Asian Americans via social support (42). Social support has been reported to serve as a protective factor for DD (43). Bangladeshi American households tend to be the most “family-based” (multigenerational families living together), when compared to all other Asian American subgroups (44), suggesting that Bangladeshis in this sample may have more sources of social support, which may confer them to be more resilient to distress when compared to non-Bangladeshis in the sample.

Those with lower emotional support had greater odds of high DD overall as well as regimen-related distress. Having confident relationships and perceived feelings of being cared for by one’s family and friends can affect one’s ability to manage T2D (45). One study found that providing emotional support via phone calls or text messages prompted better self-management of T2D for individuals living with T2D (46). Another study showed that enhancing emotional support can be a useful strategy to help reduce the burden of diabetes distress and encourage better self-management of diabetes (47).

Participants with elevated HbA1c levels had increased odds of regimen-related distress, and participants with any days of poor mental health had increased odds of overall, emotional, and physician-related distress. A San Francisco study among adult T2D patients found increases in distress was associated with poorer HbA1c outcomes (18). A scoping review indicated a significant association between HbA1c levels and DD among South Asians in low- and middle-income countries (19). Additionally, participants with any days of poor mental health may have existing mental health challenges such as depression or anxiety, making them more vulnerable to DD; several studies have shown a significant correlation between DD and depression and anxiety (26,35,48), which further explains why participants with any days of poor mental health may have increased odds of DD. Our findings suggest that South Asians with elevated HbA1c levels and any days of poor mental health may be particularly vulnerable to DD. Identifying and treating these risk factors may help decrease the burden of DD among South Asians with T2D; in addition, these groups may be particularly important to include in diabetes management efforts.

Individuals with less than a high school education had higher odds of having emotional burden distress compared to those with a college education. Education attainment is an important social determinant of health, where low education attainment has been linked to health inequities (49). A study in Pakistan reported similar findings related to education; a higher emotional burden was found to be significantly associated with low education (17). Another study on T2D patients in Saudi Arabia indicated that less education is also associated with more distress (36). Lower educational attainment has been associated with decreased health literacy and poorer health outcomes (50), which may negatively impact one’s ability to manage diabetes, leading to the observed association between low educational attainment and higher emotional burden and regimen-related distress. Finally, one study revealed that those with more education had better awareness of health complications caused by diabetes and had a higher rate of adherence to a healthy diet (51).

Data from the 2021 American Community Survey in NYC found that adults who have not graduated from high school had a higher proportion of uninsured individuals when compared to individuals with a Bachelor’s degree or higher (52); this suggests that individuals with less education may be more likely to lack health insurance, weakening their ability to consult a physician or afford diabetes medication. This may potentially lead to an increased emotional burden. A qualitative study evaluating patients’ perspectives on DD found patients were dissatisfied with diabetes care due to insurance problems. More specifically, one participant claimed, “*When I went away last week, I almost didn’t have enough medicine to go with me and the insurance won’t pay. It’s stressful*” (53). Another qualitative study reported that diabetes burnout may be linked to distress, leading to suboptimal self-management and poor diabetes outcomes (54).

### Study Limitations

Although this is one of the first studies to examine the prevalence of DD among South Asians in the US, some limitations must be acknowledged. First, measures were collected by CHWs, which may lead to response bias. Although CHWs received formal training to mitigate the possibility of response bias, participants may have felt the need to respond with positive answers. Second, this was a cross-sectional, observational study design, and therefore while covariate adjustment was included in models, causality of relationships is limited. Future research should strive to sample a larger, more varied population of South Asians. Finally, though the study recruited South Asians from different ethnic backgrounds, due to sample size constraints, the study was not able to further disaggregate data by specific country of birth or ethnicity. As our study was conducted in a predominantly immigrant, South Asian population, findings may not be generalizable to other minority communities.

## Conclusions

Our study shows that South Asians who are female, less educated, have higher HbA1c levels, and/or have poor mental health days were more likely to have higher DD. These findings contribute to current literature by highlighting the prevalence of DD among South Asians in NYC and identifying risk factors associated with DD, further endorsing that DD is a medically relevant issue that impacts the ability of South Asians to successfully manage T2D. Interventional studies as well as health policies should consider increasing the screening of DD in patients with prediabetes/diabetes and providing integrated mental/physical health services during PCP visits. Future research can also benefit from a longitudinal analysis of the impact of DD on diabetes self-management, and mental and physical health, which can help evaluate the effectiveness of potential interventions and identify possible causal relationships that may exist between DD and sociodemographic characteristics and clinical measures. Diabetes is a growing health issue in the US, and the medical and mental health needs of patients must be addressed simultaneously.

## Figures and Tables

**Table 1:** Baseline characteristics of the sample, overall and stratified by sex (n = 41)

	Overall (n = 415)		Female (n = 221)		Male (n = 194)		
	n	%	n	%	n	%	p-value
Age							
Median (IQR Range)	56.0 (48.0, 62.0)		55.0 (48.0, 61.0)		58.0 (49.0, 64.0)		0.006
Education							< 0.001
High school or less	262	63.4	168	76.7	94	48.5	
Greater than high school	151	36.6	51	23.3	100	51.5	
Marital status							< 0.001
Married	385	93.2	194	88.6	191	98.5	
Divorced/Widowed/Separated	28	6.8	25	11.4	3	1.5	
English fluency							< 0.001
Very well/Well	164	39.8	59	27.1	105	54.1	
Not well/Not at all	248	60.2	159	72.9	89	45.9	
Country of birth (Bangladesh vs. other country)							0.826
Bangladesh	286	69.4	151	68.9	135	69.9	
India	60	14.6	31	14.2	29	15.0	
Pakistan	33	8.0	22	10.0	11	5.7	
Other (Guyana, Nepal, Trinidad)	33	8.0	15	6.8	18	9.3	
Years in the US							
Median (IQR)	12.5 (7.0, 20.0)		11.0 (5.6, 19.0)		15.0 (8.0, 22.8)		< 0.001
Mental Health Days							0.007
No days	337	86.0	167	81.5	170	90.9	
Any days	55	14.0	38	18.5	17	9.1	
Physical Health Days							0.004
No days	327	83.0	161	77.8	166	88.8	
Any days	67	17.0	46	22.2	21	11.2	
Emotional Support t-score							
Median (IQR)	55.6 (47.2, 62.0)		50.8 (43.7, 62.0)		62.0 (49.0, 62.0)		< 0.001
HbA1c							
Median (IQR)	7.9 (7.4, 9.0)		7.9 (7.4, 9.2)		8.0 (7.4, 8.9)		0.820
Weight							
Median (IQR)	158.0 (139.0, 175.0)		147.0 (131.8, 16.5)		167.0 (152.0, 184.0)		< 0.001
BMI							
Median (IQR)	27.1 (24.5, 29.5)		27.4 (24.9, 31.0)		26.7 (24.3, 28.9)		0.027
SBP							
Median (IQR)	127.0 (119.0. 136.0)		125.0 (118.0, 134.0)		130.0 (120.0, 137.0)		0.012
DBP							
Median (IQR)	79.0 (73.3, 84.0)		80.0 (75.0, 83.0)		78.0 (72.0, 85.0)		0.183
BP Control (< 130/80)							0.963
Controlled	167	40.5	89	40.6	78	40.4	
Uncontrolled	245	59.5	130	59.4	115	59.6	
BP Control (< 140/90)							0.944
Controlled	340	82.5	181	82.6	159	82.4	
Uncontrolled	72	17.5	38	17.4	34	17.6	
**Diabetes Distress Scales**							
DDS Total, Continuous							
Median (IQR)	1.6 (1.3, 2.5)		1.7 (1.4, 2.7)		1.5 (1.0, 2.2)		< 0.001
DDS Total, Categories							0.031
Low (< 3)	332	84.1	167	80.3	165	88.2	
High (≥ 3)	63	15.9	41	19.7	22	11.8	
Emotional Burden, Continuous							
Median (IQR)	1.7 (1.3, 3.0)		2.0 (1.3, 3.7)		1.7 (1.0, 27)		< 0.001
Emotional Burden, Categories							0.058
Low (< 3)	304	74.1	154	70.3	150	78.5	
High (≥ 3)	106	25.9	65	29.7	41	21.5	
Physician-Related Distress, Continuous							
Median (IQR)	1.0 (1.0, 1.8)		1.3 (1.0, 1.8)		1.0 (1.0, 1.8)		0.003
Physician-Related Distress, Categories							0.488
Low (< 3)	380	93.4	199	92.6	181	94.3	
High (≥ 3)	27	6.6	16	7.4	11	5.7	
Regimen-Related Distress, Continuous							
Median (IQR)	2.0 (1.0, 2.8)		2.0 (1.5, 3.0)		1.5 (1.0, 2.3)		< 0.001
Regimen-Related Distress, Categories							0.021
Low (< 3)	318	77.8	159	71.9	159	82.8	
High (≥ 3)	91	22.2	58	26.2	33	17.2	

DDS, Diabetes Distress Scale; BP, Blood pressure; SBP, Systolic blood pressure; DBP, Diastolic blood pressure; IQR, Interquartile Range; SD, standard deviation; US, United States

**Table 2:** Unadjusted Univariate Regression Models predicting high risk DDS subscales

	DDS score - overall ≥ 3			DDS score - emotional burden ≥3			DDS score - physician-related distress ≥ 3			DDS score - regimen-related distress ≥ 3		
	OR	95% CI	p-value	OR	95% CI	p-value	OR	95% CI	p-value	OR	95% CI	p-value
**Sex (Ref = Male)**												
Female	1.8	1.1, 3.2	**0.033**	1.5	1.0, 2.4	**0.063**	1.3	0.6, 2.9	0.489	1.77	1.09, 2.86	**0.020**
**Age, continuous**	1.03	1.00, 1.06	**0.035**	1.04	1.02, 1.07	**0.001**	1.02	0.97, 1.06	0.469	1.02	0.99, 1.04	0.240
**Education (Ref = College graduate)**												
Less than high school	19.9	4.7, 84.0	**< 0.001**	15.5	6.0, 40.1	**< 0.001**	2.8	0.9, 8.4	**0.072**	9.4	3.9, 22.6	**< 0.001**
High school/Some college	3.1	0.6, 14.9	**0.160**	2.6	0.9, 7.4	**0.065**	0.7	0.2, 2.9	0.647	2.1	0.8, 5.4	**0.150**
**Marital Status (Ref = Not married)**												
Married	4.6	0.6, 34.4	**0.141**	1.2	0.5, 3.1	0.687	0.9	0.2, 4.1	0.917	2.2	0.6, 7.4	0.217
**English Fluency (Ref = very well/ well)**												
Not well/Not at all	3.7	1.9, 7.3	**< 0.001**	2.6	1.6, 4.3	**< 0.001**	1.4	0.6, 3.1	0.459	2.2	1.3, 3.6	**0.003**
**Country of birth (Ref = Other Country)**												
Bangladesh	170.1	0.0, 0.1	**< 0.001**	0.1	0.0, 0.1	**< 0.001**	0.3	0.1, 0.6	**0.002**	0.1	0.1, 0.2	**< 0.001**
**Years in the US, continuous**	1.02	0.99, 1.05	**0.182**	1.04	1.02, 1.07	**0.001**	0.99	0.95, 1.03	0.627	1.02	0.99, 1.04	0.189
**Mental Health Days (Ref = No days)**												
Any days	2.0	1.0, 4.1	**0.048**	1.8	1.0, 3.4	**0.049**	3.8	1.5, 9.4	**0.004**	1.4	0.7, 2.7	0.301
**Physical Health Days (Ref = No days)**												
Any days	0.8	0.4, 1.8	0.621	1.0	0.6, 1.9	0.884	1.9	0.8, 4.7	**0.170**	0.8	0.4, 1.6	0.608
**Emotional support t-score, continuous**	0.88	0.85, 0.92	**< 0.001**	0.92	0.89, 0.94	**< 0.001**	0.97	0.93, 1.01	**0.109**	0.91	0.89, 0.94	**< 0.001**
**BMI, continuous**	1.00	0.95, 1.06	0.998	1.02	0.97, 1.06	0.494	1.01	0.94, 1.10	0.746	1.04	0.99, 1.09	**0.098**
**Weight, continuous**	1.00	0.99, 1.01	0.696	1.01	1.00, 1.01	0.213	1.00	0.99, 1.02	0.786	1.01	1.00, 1.02	**0.085**
**HbA1c, continuous**	1.31	1.10, 1.56	**0.003**	1.20	1.03, 1.40	**0.019**	1.15	0.89, 1.49	0.278	1.36	1.16, 1.59	**< 0.001**
**SBP, continuous**	1.02	1.00, 1.04	**0.070**	1.01	1.00, 1.03	**0.121**	0.99	0.96, 1.02	0.468	1.01	1.00, 1.03	**0.174**
**DBP, continuous**	1.01	0.97, 1.04	0.776	1.01	0.98, 1.03	0.671	0.97	0.92, 1.01	**0.168**	1.00	0.97, 1.03	0.947
**BP Control (< 140/90) (Ref = Controlled)**												
Uncontrolled	1.4	0.7, 2.8	0.292	1.4	0.8, 2.4	0.249	0.4	0.1, 1.5	**0.168**	1.3	0.7, 2.4	0.349

BP, Blood Pressure; DDS, Diabetes Distress Scale; SBP, Systolic Blood Pressure; DBP, Diastolic Blood Pressure; Body mass index, BMI

**Table 3:** Adjusted Regression Model predicting high risk DDS subscales

	DDS score - overall ≥ 3			DDS score - emotional burden ≥3			DDS score - physician-related distress ≥ 3			DDS score - regimen-related distress ≥ 3		
	OR	95% CI	p-value	OR	95% CI	p-value	OR	95% CI	p-value	OR	95% CI	p-value
**Sex (Ref = Male)**												
Female	1.0	0.4, 2.2	0.975	1.4	0.7, 2.7	0.302	0.8	0.3, 2.2	0.840	1.2	0.6, 2.2	0.648
**Age, continuous**	1.01	0.97, 1.06	0.648	1.03	1.00, 1.07	0.077	1.00	0.96, 1.06	0.869	0.99	0.96, 1.03	0.992
**Education (Ref = College graduate)**												
Less than high school	3.8	0.7, 20.6	0.122	4.5	1.5, 13.6	**0.007**	1.3	0.3, 5.0	0.728	2.8	1.0, 7.7	0.052
High school/Some college	3.1	0.5, 17.2	0.202	2.4	0.7, 7.2	0.136	0.7	0.2, 3.1	0.647	1.8	0.6, 4.3	0.417
**Country of birth (Ref = Other Country)**												
Bangladesh	0.1	0.0, 0.3	**< 0.001**	0.1	0.0, 0.2	**< 0.001**	0.3	0.1, 0.8	**0.015**	0.2	0.1, 0.3	**< 0.001**
**Years in US, continuous**	0.99	0.94, 1.03	0.499	1.02	0.99, 1.06	0.176	0.98	0.93, 1.04	0.514	0.99	0.96, 1.03	0.630
**Mental Health Days (Ref = No days)**												
Any days	3.7	1.3, 10.4	**0.014**	4.9	2.1, 11.3	**< 0.001**	5.0	1.8, 13.7	**0.002**			
**Emotional support t-score**	0.92	0.88, 0.96	**< 0.001**							0.95	0.92, 0.99	**0.006**
**HbA1c, continuous**	1.26	1.00, 1.60	0.052							1.31	1.08, 1.60	**0.007**

BP, Blood Pressure; DDS, Diabetes Distress Scale; DBP, Diastolic Blood Pressure; SBP, Systolic Blood Pressure

## Data Availability

The data and materials generated during this study are not publicly available, as the study is ongoing, but will be available from the corresponding author upon reasonable request.

## Abbreviations

BMI: Body mass index
CHW: Community health worker
CI: Confidence interval
DD: Diabetes Distress
DDS: Diabetes Distress Scale
DREAM: Diabetes Research, Education, and Action for Minorities Initiative
DBP: Diastolic blood pressure
EHR: Electronic health record
HbA1c: Hemoglobin A1c
IQR: Interquartile range
NHANES: National Health and Nutrition Examination Survey
NYC: New York City
NYC CHS: NYC Community Health Survey
NYC HANES: NYC Health and Nutrition Survey
OR: Odds ratio
PCP: Primary care practice
REACH: Racial and Ethnic Approaches to Community Health
SBP: Systolic blood pressure
T2D: Type 2 diabetes
US: United States
